# DeepMiR2GO: Inferring Functions of Human MicroRNAs Using a Deep Multi-Label Classification Model

**DOI:** 10.3390/ijms20236046

**Published:** 2019-11-30

**Authors:** Jiacheng Wang, Jingpu Zhang, Yideng Cai, Lei Deng

**Affiliations:** 1 School of Computer Science and Engineering, Central South University, Changsha 410083, China; jiachengwang@csu.edu.cn (J.W.); iamcyd2017@csu.edu.cn (Y.C.); 2 School of Computer and Data Science, Henan University of Urban Construction, Pingdingshan 467000, China; zhangjp@csu.edu.cn; 3 School of Software, Xinjiang University, Urumqi 830008, China

**Keywords:** MicroRNA function, heterogeneous network, graph embedding, deep multi-label classification

## Abstract

MicroRNAs (miRNAs) are a highly abundant collection of functional non-coding RNAs involved in cellular regulation and various complex human diseases. Although a large number of miRNAs have been identified, most of their physiological functions remain unknown. Computational methods play a vital role in exploring the potential functions of miRNAs. Here, we present DeepMiR2GO, a tool for integrating miRNAs, proteins and diseases, to predict the gene ontology (GO) functions based on multiple deep neuro-symbolic models. DeepMiR2GO starts by integrating the miRNA co-expression network, protein-protein interaction (PPI) network, disease phenotype similarity network, and interactions or associations among them into a global heterogeneous network. Then, it employs an efficient graph embedding strategy to learn potential network representations of the global heterogeneous network as the topological features. Finally, a deep multi-label classification network based on multiple neuro-symbolic models is built and used to annotate the GO terms of miRNAs. The predicted results demonstrate that DeepMiR2GO performs significantly better than other state-of-the-art approaches in terms of precision, recall, and maximum F-measure.

## 1. Introduction

MicroRNAs (miRNAs) are an abundant collection of short non-coding RNAs encoded by endogenous genes about 20∼25 nucleotides in length [1]. They play vital roles in regulating mRNA translation and suppressing post-transcriptional modification in human beings and other organisms by base-pairing with mRNA molecules [2]. Since researchers reported lin-4 and let-7 RNAs encoded in worms and mammals, thousands of miRNAs have been discovered in a multitude of organisms [3,4,5]. The number of identified miRNAs has increased significantly in recent years, with a massive amount of research focused on this field [6,7]. Accumulating evidence demonstrates that miRNAs are involved in numerous biological processes, including regulation of cell proliferation, cell death, development, differentiation, and immune reactions [1,8]. Numerous studies have indicated that miRNAs are also related to various diseases [9,10,11,12]. However, no method can detect the function of miRNAs at a large scale, and the functions of most miRNAs remain unknown. Thus, the annotation of unknown miRNA functions has become a research hotspot within biology and bioinformatics.

Upregulation or downregulation analysis of high-throughput miRNA expression profiles is primarily employed to identify the functions of miRNAs [13]. Lu et al. adopted an miRNA expression profiling method based on beaded flow cytometry to perform systematic expression analysis on mammalian miRNA and found a general downregulation of miRNA in tumours. In addition, building the genome-wide interaction maps of molecules for analysis of miRNA mechanisms is also an indispensable approach for high-throughput sequencing technology [14]. The molecular functions of miRNAs can be regarded as multi-type functions of the interactions between miRNAs and other biological molecules (e.g., DNAs, proteins, RNAs, etc.). Focusing on exploring RNA-DNA interactions, various experimental methods have been proposed, such as capture hybridization analysis of RNA targets (CHART) developed by Simon et al. [15]. In addition to DNA, RNA-protein interactions generally play a vital role in a substantial number of cellular processes [16,17]. Combining those interactions is usually necessary to investigate the functions of miRNAs. However, identifying functions of miRNA by experimental approaches is considerably expensive and progressing slowly.

With abundant useful data regarding the produced miRNAs, many computational methods have been proposed for inferring miRNA function. Some approaches, based on the theory that genes with similar expression schemas across various different tissues tend to share identical or similar biological functions [18,19], analyse the miRNA co-expression patterns to investigate their functional roles [20]. In addition, many researchers focused on elucidating miRNA functions through target gene prediction in view of the relations between functions of miRNA and functions of their target gene production [21,22]. Benjamin et al. designed a tool called TargetScan [23]. TargetScan combined sequence comparison analysis with the thermodynamic model of RNA-RNA duplex interactions to infer the mRNAs of vertebrate miRNAs, and predicted diverse molecular functions of 121 miRNAs by assessing target gene functions. DIANA-microT [24], a tool developed by Maragkakis et al. to predict target genes of miRNA, further elucidated miRNA functions through analysing Kyoto Encyclopedia of Genes and Genomes (KEGG) pathways of predicted targets. However, there are two critical challenges for the above methods. First, because the majority of the predicted target genes of miRNAs are negative, the predictive performances are not very reliable. Second, the methods based on predicting target genes of miRNAs overlook other important biological data, such as the expression profiles of miRNAs [25]. Hence, the performances of these tools are not satisfactory or reliable. Backes et al. [26] proposed an efficient approach, called miEAA, to infer functions of miRNA based on enrichment analysis. However, a significant challenge to miEAA is that miRNAs usually do not function in isolation. In general, one miRNA may act on numerous target genes, and one target gene may be regulated by many miRNAs together [27,28]. The potential associations between miRNAs and co-expression patterns also play an essential role in understanding the biological mechanisms and inferring the physiological functions of miRNAs. Moreover, miEAA ignores the significant associations of miRNAs with proteins and diseases, which provide sufficient latent information for annotating miRNAs. To address these challenges, we recently designed a novel tool, named PmiRGO [29], to predict the gene ontology (GO) terms of miRNAs on a large scale by integrating multiple biological molecule networks, including the miRNA co-expression similarity network, protein-protein interaction (PPI) network, and their interaction network.

In this paper, we propose a novel approach, called DeepMiR2GO, which utilizes multiple deep neuro-symbolic models to annotate gene ontology (GO) functions of miRNAs at a large scale. First, we build a global miRNA-protein-disease network by combining three biological entity networks, including miRNA co-expression similarity network, PPI network, and disease similarity network, via interaction or association networks among them. Secondly, we apply an efficient graph embedding algorithm, LINE [30], to generate low-dimensional and higher-order structural features of the heterogeneous network. Finally, we construct a deep neuro-symbolic model for every GO result that resembles dependencies and the structure among GO terms and annotates GO functions of miRNAs over the whole ontology hierarchy by utilizing structural features to refine representations and predictions for each level of GO [31]. DeepMiR2GO achieves a maximum F-measure of 0.399 on the independent miRNA2GO-337 dataset and demonstrates that DeepMiR2GO significantly outperforms two state-of-the-art computational approaches: miEAA and PmiRGO.

## 2. Results

### 2.1. Benchmarks

To assess the predictive performance of DeepMiR2GO more accurately, we built a benchmark including 337 mature miRNAs (named as miRNA2GO-337) based on the GOA database [32,33]. In miRNA2GO-337, each miRNA has at least one manually curated GO annotation supported by non-IEA evidence. The miRNA2GO-337 dataset appears in Supplementary Table S1.

### 2.2. Parameter Selection

Here, we discuss the parameter settings for our model. There are several hyper-parameters necessary to be optimized in the step of learning network topological features. Among these parameters, the dimension of features has a significant influence on the performance of the prediction of miRNAs. To evaluate the effect of the hyper-parameters on the predictive performance, we vary the number of dimensions and carry out an independent test on the benchmark. For the other parameters, including the starting value $p_{0}$ of the learning rate, the number of negative samples *n*, and the total number of samples *T*, we selected the values of the three parameters by executing experiments with different values and screening out the combination which performed best ($p_{0}=0.025$, n=10, T=100 billion).

Table 1 demonstrates the effects of the dimension of network features, which ranges from 64 to 512. Generally speaking, effective feature selection algorithms can eliminate irrelevant or redundant features to obtain the optimal feature set, thereby improving the performance of predictive model [34,35,36]. In our work, the network features we obtained contain local and global information of each node about a certain context in the heterogeneous biological graph. In order to preserve these local and global information, we did not apply the feature selection process. As shown, the maximum F-measure reaches the highest BPs (biological processes) when the number of dimensions is set to 64. Therefore, 64-dimensional features of the network are selected in our work.

For the parameters of the hierarchical neuron network model, including the amount of neurons in the fully connected layer, starting learning rate and minibatch size, we manually tuned them and chose the combination of parameters with the best evaluated performance (minibatch size = 64, amount of neurons in fully connected layer = 1024, starting learning rate = 0.025).

### 2.3. Incorporating Disease Similarity Network

Increasing evidence suggests that the majority of miRNAs have many vital associations with various diseases, including cardiovascular diseases, schizophrenia and cancer [37,38]. In addition, the dysregulation of miRNA target genes may also lead to many kinds of diseases. We reasoned that introducing disease entities would be an efficient approach to significantly improve the performance of prediction. In our work, we incorporated disease phenotype similarity network into our miRNA-protein entities network. To evaluate the effect of incorporating disease similarity network and validate our deduction, we trained our model with global heterogeneous networks with and without disease entities, respectively, and then evaluated both on the independent test set miRNA2GO-337. In the set of comparative experiments, all of the parameters were the same. The results are shown in Table 2. The predictive performance with disease entities is significantly better than the performance without disease entities among all three measurements, with the maximum F-measure increased by 4% on BP and 23% on MF (molecular function). The results demonstrated that incorporating the disease entity network significantly improves the predictive performance of the miRNA gene ontology function for almost all GO classes.

### 2.4. Comparison of Three Classic Network Representation Algorithms

Recently, vast research efforts have proven that representation learning for networks can produce reasonably effective features for common tasks of machine learning on graphs, such as multi-label classification [39], tag recommendation [40], and link prediction [41,42]. Most of these studies primarily investigated latent topological information associated with each vertex from the network structure. A recently proposed approach for graph representation learning, DeepWalk, truncates random walks starting from each node to extract the contextual information based on a neural network model [43]. On this basis, node2vec improves the random walk phase and combines BFS-like and DFS-like neighbourhood investigations to obtain different network structure information by introducing a return parameter *p* and an in-out parameter *q* [44]. Moreover, a novel network embedding approach, named LINE, was developed for large-scale networks. LINE optimizes an objective function which synthesizes the first-order and second-order approximations to extract the local and global topological network structure [30].

**Figure 1:**
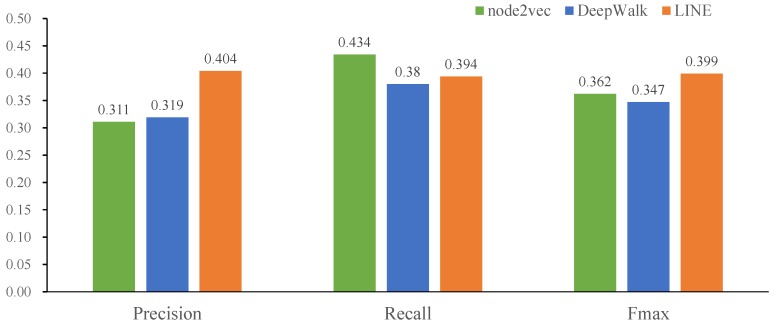
Performance comparison among three network representation learning approaches (DeepWalk, node2vec, and LINE). The predictive performances of the three approaches were evaluated on the miRNA2GO-337 dataset.

In this paper, three network representation algorithms were applied to obtain the topological feature vectors of the same global heterogeneous network, respectively. Then, we utilized the hierarchy multi-classification model for training and compared the performances of three network embedding models on the miRNA2GO-337 dataset. Figure 1 shows that LINE achieves the highest maximum F-measure and average precision on BP, while DeepWalk and node2vec outperform LINE on MF.

### 2.5. The Effect of Hierarchical Multiple Classification Model

In our work, we built a deep hierarchical multi-label classification model to infer probable functions of miRNAs on a large scale. The deep classification model can optimize the predictive performance of GO functions through utilizing the hierarchy of GO and their dependencies between its terms. To evaluate the effect of the deep classification model, we also selected three conventional machine learning methods, including SVM [45,46,47], decision tree [48], and random forest [49,50], that are well known for good effects on the multi-class issue, as the base multi-label classifier and compared their predictive performances. These classic machine learning methods have been successfully applied in the field of bioinformatics to solve various problems, such as the prediction of therapeutic peptides used to treat cancers and autoimmune diseases [51,52,53,54]. In this study, the training data, test data, and the network features used in the three base classifiers are the same as in our deep classification model. We optimized the penalty parameter *C* and the kernel parameter γ of SVM by grid search method. The hyper-parameters of decision tree and random forest, including the maximum depth of the tree and the minimum number of samples at a leaf node, were optimized by the same way as well as SVM. Furthermore, we applied a 10-fold cross validation to evaluate the performance of each combination of these hyper-parameters for the three base methods. These hyper-parameters of SVM, decision tree and random forest were tuning in the following search range, respectively.

(1) $$\left\{\begin{array}{c} 2^{-5}\leq C\leq 2^{15},\;step=2\\ 0\leq \gamma \leq 2,\;\;\;step=2^{-5} \end{array}\right.$$

(2) $$\left\{\begin{array}{c} 3\leq MaxDepth\leq 13,\;\;\;\;step=1\\ 1\leq MinSamplesLeaf\leq 10,\;step=1 \end{array}\right.$$

(3) $$\left\{\begin{array}{c} 3\leq MaxDepth\leq 13,\;\;\;\;step=1\\ 1\leq MinSamplesLeaf\leq 10,\;\;step=1\\ 100\leq NumberofTrees\leq 500,\;step=50 \end{array}\right.$$

**Figure 2:**
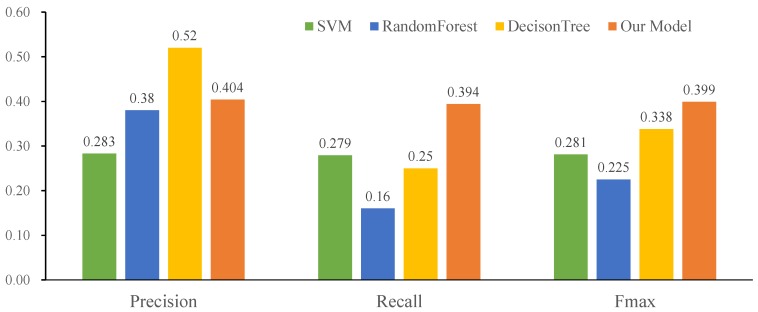
Performance comparison of different multi-label classification models (SVM, decision tree, random forest and our model) on the miRNA2GO-337 dataset.

Figure 2 displays the predictive performances of our deep hierarchical multi-label classification model, as well as those of the base multi-label classifiers. Compared with SVM and Random Forest, our classification model performs better in terms of all metrics. Moreover, our model achieves the highest $F_{max}$, 0.399, among all of the classification models.

### 2.6. Performance

For further evaluation of performance, we compared our DeepMiR2GO model with two state-of-the-art approaches, miEAA [26] and PmiRGO [29], on the independent test set miRNA2GO-337. Backes et al. designed miEAA based on GeneTrail, a statistical framework of the gene set analysis toolkit [55]. miEAA offers the functional analysis of precursor miRNAs as well as mature miRNAs by utilizing enrichment analysis. In miEAA, 14,000 different miRNA sets from various common miRNA databases and academic literature were collected and integrated to provide a wide range of functions and applicability. PmiRGO is an approach based on traditional machine learning. It combines multiple networks, including the miRNA co-expression similarity, miRNA-target gene interaction and PPI networks, and then applies the multi-classification model based on SVM to predict the gene ontology functions of miRNAs. As PmiRGO only performs on BP, we carried out the comparison in terms of BPs.

**Figure 3:**
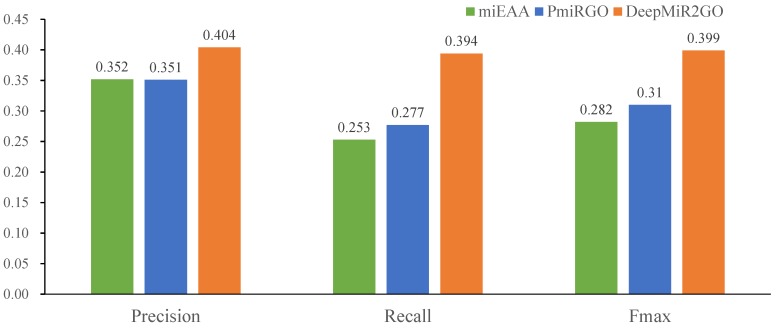
Performance comparison among miEAA, PmiRGO, and DeepMiR2GO in terms of precision, recall, and Fmax. The predictive performances of the three methods wew evaluated on the miRNA2GO-337 dataset.

**Figure 4:**
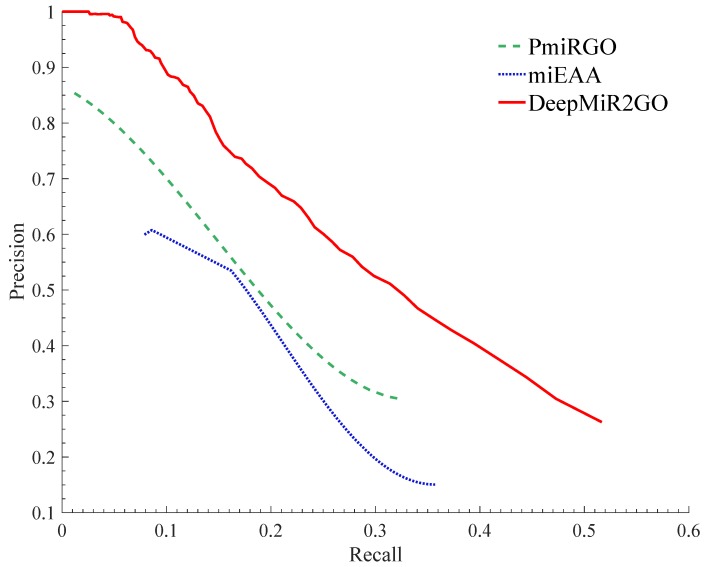
Precision-recall (P-R) curves of miEAA, PmiRGO, and DeepMiR2GO. The performances of the three methods were evaluated on the miRNA2GO-337 dataset in terms of BPs (biological processes).

The predictive performance comparison among DeepMiR2GO and the two other approaches is shown in Figure 3. Our DeepMiR2GO significantly outperforms miEAA and PmiRGO in terms of all measures. For the maximum F-measure metric, DeepMiR2GO achieved 0.399 on BP with increases of 11.7% and 8.9%, respectively, while miEAA and PmiRGO achieved values of 0.282 and 0.31. In terms of average precision and recall, our method is also better than miEAA and PmiRGO, and reached 0.404 and 0.394, respectively. Moreover, we present the precision-recall curves of our method as well as two state-of-the-art methods. As shown in Figure 4, the P-R curve of DeepMiR2GO is far above PmiRGO and miEAA, further demonstrating that our model performs much better than PmiRGO and miEAA.

**Figure 5:**
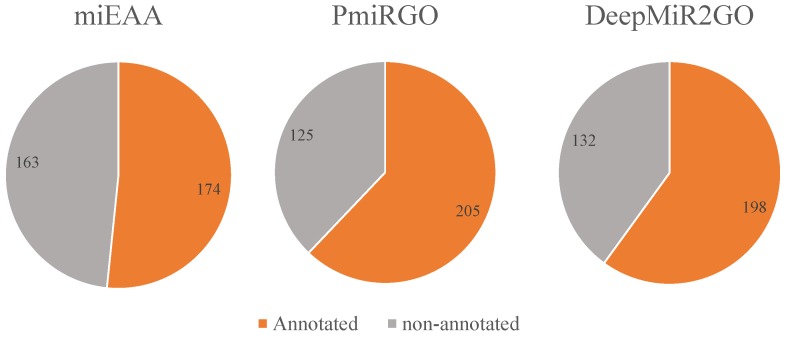
Performance comparison of coverage among miEAA, PmiRGO, and DeepMiR2GO on the independent dataset miRNA2GO-337.

In addition, we also compared the coverage on the miRNA2GO-337 dataset among the three methods through counting the number of miRNAs predicted with at least one BP GO term. As depicted in Figure 5, DeepMiR2GO correctly predicted 198 miRNAs out of 337 miRNA samples, much higher than miEAA and slightly lower than PmiRGO, which proves that DeepMiR2GO achieves good coverage.

### 2.7. Case Study

To further verify the predictive performance and widespread applications of our approach, DeepMiR2GO was applied to infer the GO functions of two miRNAs in the independent dataset miRNA2GO-337 as instances: hsa-miR-92a-3p and hsa-miR-138-5p. The mature miRNA hsa-miR-92a-3p forms from two hairpin precursor miRNAs: hsa-miR-92a-1 on chromosome 13 and hsa-mir-92a-2 on chromosome X. Researchers have demonstrated that hsa-miR-92a-3p regulates the development and homoeostasis of cartilage by evaluating the invitro expression of hsa-miR-92a-3p in a human mesenchymal stem cell (hMSC) model of chondrogenesis [56]. Sharifi et al. [57] applied cell proliferation and phase-locking nucleic acid (LNA) to block hsa-miR-92a-3p in a human acute megaloblastic leukaemia cell line (M-07e) and found that it plays an important role in regulating the viability of M-07e cells. Ma et al. [58] used real-time quantitative polymerase chain reaction (RT-qPCR) and identified aberrantly expressed hsa-miR-92a-3p in schizophrenia, which revealed that hsa-miR-92a-3p has essential roles in the context of schizophrenia. Moreover, researchers have demonstrated that the secretion of hsa-miR-92a-3p by liposarcoma cells promotes the proliferation, invasion and metastasis of liposarcoma cells through this interaction with the surrounding microenvironment by extracellular vesicles and through stimulation of the secretion of the pro-inflammatory cytokine interleukin 6 in the TLR7/8-dependent manner of tumour-related macrophages [59].

We apply DeepMiR2GO to predict the GO classes of hsa-miR-92a-3p. The GO terms predicted are listed in Table 3, and most of them are associated with macromolecule metabolic processes and cellular processes. The previous experiments have validated these physiological functions. The predicted results of hsa-miR-92a-3p demonstrate that DeepMiR2GO can automatically annotate the ontology functions of miRNAs with high accuracy.

To further evaluate the predictive performance about the precise terms, we use DeepMiR2GO to predict the GO terms of hsa-miR-138-5p. The top 10 GO terms predicted, as well as their depth in the DAG (directed acyclic graph) of GO, are list in Table 4. Most of them are more than 8 layers deep, and the deepest term is in the 13th layer. Among these predicted GO terms, GO:0045944, GO:0010629 and GO:0008285 have been experimentally verified. The results demonstrate that DeepMiR2GO can predict very specific biological functions of miRNAs.

## 3. Discussion

A variety of computational approaches have been developed to predict the biological functions of microRNA in the last dozen years. Among these approaches, the most basic strategy is to first predict the target genes of miRNA and then to infer the ontological functions of miRNA through analysing ontological functions of its target genes [24]. However, the performance of this strategy is directly affected by the tools used to predict target genes. Methods based on the analysis of miRNA expression profiles adopt a more direct approach to identify the functions of miRNA and can perform better than those target gene prediction tools. In addition, some deep-level physiological functions are not able to be determined from the analysis of a single miRNA’s expression profile but require more interaction or association information, all of which contribute much to the ontological functions and biological processes of miRNA. To address this problem, Deng et al. [29] introduced protein-protein interaction networking by connecting with the miRNA co-expression network to learn potential topological information between miRNA and protein entities or significant network patterns that are considered quite useful to predict the functions. In this work, we take the important associations between miRNA and disease into consideration and introduce disease entities into our research. Three biological entity networks, including the miRNA co-expression network, PPI network, and disease similarity network, and the association networks among them, are integrated into a global heterogeneous network.

Furthermore, many researchers have applied network representation learning to extract effective structural features of a large information network, which are very useful in many challenging tasks such as visualization, node classification, tag recommendation, and link prediction [60]. Various network representation learning algorithms have been proposed to address machine learning issues and have proven to be significantly effective. In our work, we also employed a classic and efficient method, called LINE [30], to learn some latent topology information and obtain low-dimensional network representations of the global heterogeneous network.

Furthermore, the deep hierarchical multi-label classification model also contributes to our work [31]. It utilizes the hierarchy of GO and the dependencies between its terms to learn latent representations and to optimize the predictive performance on whole hierarchies in an end-to-end manner. Especially, the latent energy for end-to-end learning of the deep hierarchical model provides advantages over those methods that depend on hand-crafted feature vectors, such as structured SVM.

## 4. Materials and Methods

### 4.1. MiRNA Co-Expression Similarity Network

We downloaded the miRNA expression profiles from the miRmine database, which contains 2822 precursor miRNA expression profiles collected from different miRNA-seq datasets available to the public and substantial detailed data regarding various miRNAs [61]. Each expression profile consists of 135 columns of expression values measured from 15 types of human tissues. Generally, two or more homologous precursor miRNAs may produce a mature miRNA. In this paper, we averaged the expression profiles of different homologous precursor miRNAs to obtain the standard expression values of the mature miRNA they generated. In consequence, we obtained the expression profiles of 2588 mature miRNAs. Then, the Pearson’s Correlation Coefficient (PCC) scores of the expression profiles between each pair of miRNAs were calculated as the co-expression similarity [62]. Finally, an miRNA co-expression network was constructed based on the co-expression similarity scores. Since we used the PCC values as the weights of the edges of the network and only considered non-negative weights, the negative PCC scores were screened out in our work.

### 4.2. Protein-Protein Interaction Network

We extracted the PPI data from the STRING database V10.0, which contains known and predicted PPIs [63]. These interactions include both physical and functional associations, stemming from not only biological experiments, but also computational prediction tools and text mining approaches. Single or multiple available lines of evidence with high probability contribute to the confidence scores of each interaction. The higher the confidence score of the interaction, the more likely two proteins in the entry are to interact with each other. We then constructed a PPI network, which consists of 18,143 proteins and 7,866,428 interactions between these proteins. Moreover, we treated the confidence score as the weight of the edge in the PPI network and set the weight to 0 if two proteins have no interaction.

### 4.3. Disease Similarity Network

We obtained the disease phenotype similarity data consisting of 5080 diseases from MimMiner [64]. By text mining and analysing phenotype records in the Online Mendelian Inheritance in Man (OMIM) database utilizing Medical Subject Headings (MeSH) terms, MimMiner provides a disease similarity value for each phenotype pair. Driel et al., the developer of MimMiner, uses a weighted vector of normalized phenotypic features to characterize every phenotype. After that, the similarity score between each disease phenotype pair is generated by computing the cosine value of their eigenvector angle. In our work, the similarity score of two disease phenotypes is regarded as the weight of their edge in the disease similarity network.

### 4.4. miRNA-Target Interaction Network

The miRNA-target gene interactions data used in our work were retrieved from the miRTarBase database of release 7.0 [65]. The database contains a large number of experimentally verified miRNA-target interactions obtained by surveying pertinent literature after systematic natural language processing of the text systematically to screen out research articles relevant to functional studies of miRNAs. After removing the duplicate and out-of-range entries, 355,684 miRNA-target interactions of high quality were obtained, including 2588 miRNAs and 18,143 target genes, and we built an miRNA-target interaction network. Note that the weight of the edge of the network is set to 1 if one miRNA interacts with one protein.

### 4.5. miRNA-Disease Association Network

The human microRNA and disease associations were extracted from HMDD v3.0 [66]. The database includes 32,281 high-quality association entries among 1206 miRNAs and 893 diseases with multiple types of experiment-supported evidence. We merged the different pre-miRNAs, which generate the same mature miRNA, and converted them into mature miRNAs. Moreover, we removed those associations that were out of range of the disease similarity network and miRNA co-expression network. Finally, 11,824 miRNA-disease associations were employed in our work.

### 4.6. Gene-Disease Association Network

To obtain protein-disease associations for connecting the two biological entity networks of PPIs and disease similarity, we downloaded the gene-disease association data from the DisGeNET database, collecting data from expert-curated repositories, the scientific literature, GWAS catalogues, and animal models [67]. Additionally, this resource provides a few primary metrics to prioritize the genotype-phenotype associations. The DisGeNET database consists of 628,685 entries of gene-disease association, including 17,549 genes and 24,166 diseases. In our work, we turned genes into proteins using the mapping between them and unified the name of diseases. After removing the redundancy and out-of-range associations, a set of 87,347 protein-disease associations was retrieved and utilized to build the protein-disease association network.

### 4.7. Methods

**Figure 6:**
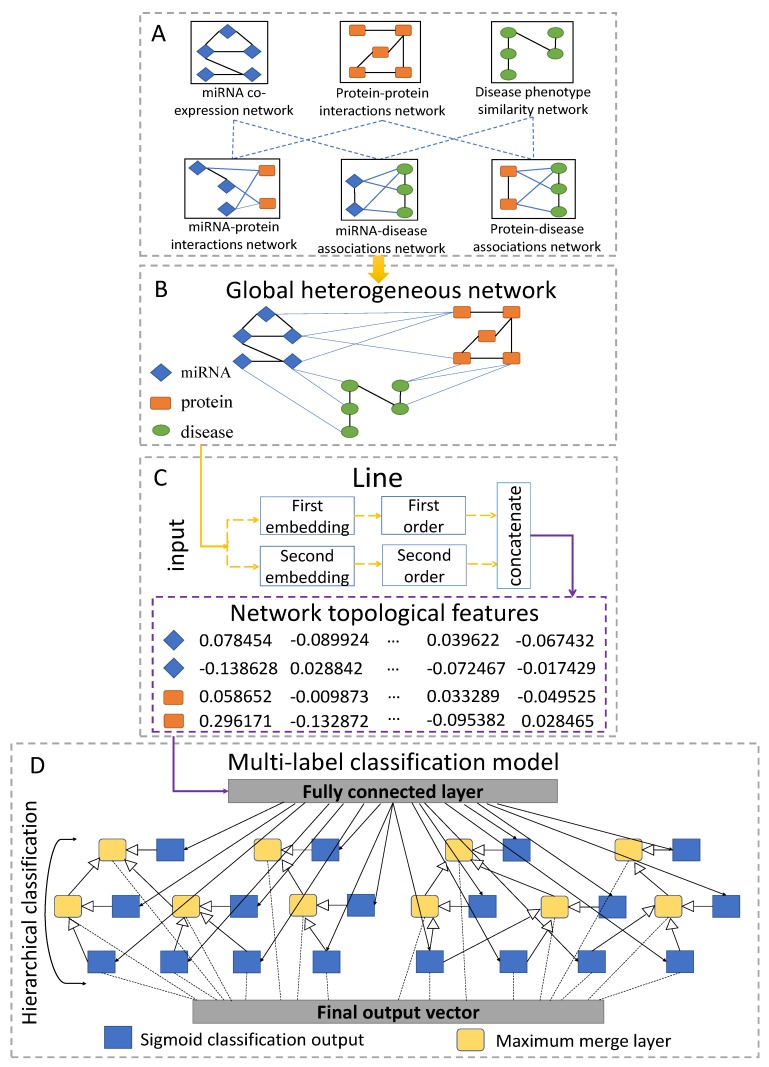
Flowchart of DeepMiR2GO. (**A**) Construct the miRNA co-expression similarity network, the PPI network, the disease similarity network, and their association networks among the three biological entities. (**B**) Build the global heterogeneous biological entity network by integrating the six networks described above. (**C**) Employ a representation learning strategy (LINE) to extract low-dimensional feature vectors of the global heterogeneous network. (**D**) Construct a deep hierarchical multi-label classification model using the feature vectors of nodes to train the model and to predict the gene ontology classes of miRNAs.

The flowchart of DeepMiR2GO is illustrated in Figure 6. There are four steps in our method: (A) construct an miRNA co-expression similarity network, a PPI network, a disease similarity network, and association networks among the three biological entities; (B) build the global heterogeneous biological entity network by integrating the six networks described above; (C) employ network representation learning to obtain the low-dimensional topological features vector for each node of the global heterogeneous network; and (D) construct a deep hierarchical multi-label classification model using the feature vectors of the nodes to train the model and predict the gene ontology functions of miRNA.

#### 4.7.1. Constructing Global Heterogeneous Network

Three biological entity networks are constructed as described in the previous chapter: the miRNA co-expression similarity network, the PPI network, and the disease phenotype similarity network. Specifically, miRNAs with identical expression patterns tend to share similar biological pathways or functions [18,68]. The Pearson’s correlation coefficient (PCC) scores of the expression profiles between each pair of miRNAs are calculated to represent the co-expression similarity. Furthermore, the similarity scores of the miRNA co-expression and disease phenotype similarity networks are treated as weights of the edges in the networks, as well as the predicted scores of the PPI network. By assembling the miRNA co-expression similarity network, PPI network, and disease phenotype similarity network and utilizing associations or interactions among the three biological entity networks, we connect them and construct a global heterogeneous network.

#### 4.7.2. Learning Topological Features

We apply a novel graph representation learning strategy, called LINE, that suites large-scale and multiple types of data networks to obtain the low-dimensional topological features vector for each node of the global heterogeneous network [30]. Given an information network, we define it as G=(V,E), where *V* represents the set of vertices and *E* indicates the set of edges between the vertices. Each edge e∈E represents a relationship between two vertices and is possessed with a weight $w_{ij}>0$, indicating the strength of the relationship. Note that the global heterogeneous network in our work is undirected, as it can be considered as a social network.

To extract sufficient topological information from the network, LINE explores not only the local but also the global network structures. Specifically, the local structures are defined as the first-order approximation between the nodes. For every undirected edge e(i,j) in the network, the weight, $w_{ij}$, representing the joint probability between node $V_{i}$ and $V_{j}$, denotes the first-order proximity. If there is no edge between two nodes, their first-order proximity is set as zero. Based on the assumption that vertices shared with neighbours have a high probability of similarity, the second-order proximity between the nodes, which measures the global structures of the network, is further explored by determination through the shared neighbourhood structures of the nodes. Mathematically, we use $Pro_{i}=\left(w_{i,1},w_{i,2},\ldots ,w_{i,|V|}\right)$ to denote the first-order proximity of $V_{i}$ with all of the other nodes, then we calculate the similarity between $Pro_{i}$ and $Pro_{j}$ as the second-order proximity $V_{i}$ and $V_{j}$. Furthermore, two models separately preserved with first-order and second-order approximations are trained and then concatenated to generate the representations of the network.

#### 4.7.3. Training the Hierarchical Multi-Label Classification Network

A common challenge to machine learning-based multi-label classification methods is the scarcity of training datasets. Moreover, approaches based on deep neural networks require far more training samples for accurate predictions. In our work, building the training datasets based on the miRNAs directly would lead to poor predictive performance because of the lack of experimentally validated GO annotations of miRNAs. Thus, we downloaded gold standard GO annotations of proteins from the GOA database (version 201604) to train our model [69]. In particular, we picked the proteins with sequence lengths of 50–100 aa and clustered them with a sequence similarity of 90 percent [70]. Then, we selected only one sample from each cluster. In addition, we screened out those samples without at least one GO term supported by non-IEA evidence (not inferred from electronic annotation). After that, 19,208 proteins as well as their GO annotations of 2342 classes were obtained. Gene Ontology consists of three branches: molecular functions (MF), biological processes (BP), and cellular components (CC).In this paper, we divided the 2342 classes of GO annotations into the three subsets: 1727 GO terms for BP, 355 GO terms for MF and 260 GO terms for CC, respectively.

As gene ontology (GO) terms are regarded as a form of hierarchical Directed Acyclic Graph (DAG), where each term is associated with one or more other terms in the same domain or different domain, the prediction of miRNA GO terms can be regarded as a hierarchical multi-classification [31]. In our work, we build a deep multi-label hierarchical classification model consisting of multiple neural-symbolic models for each class in GO, which encodes for the transitivity of subclass relations. The low-dimensional feature vectors of the global heterogeneous network are input to the hierarchical classification model and trained sequentially, level by level. For those training samples which do not have network features, we assign a vector of zeros with the same dimension. Each neural network consists of one fully connected layer and a sigmoid activation function layer. The output vectors of the first fully connected layer are input into the following layer. Note that all neurons share the fully connected layer. To ensure consistent hierarchical classification, we created a maximum merge layer for each term with child nodes in GO. The merge layer picks the maximum value from the predicted scores of the term and all of its child terms. As a result, the final output vector of the classification model is the concatenation of activation layers of leaf nodes and the maximum merge layers of non-leaf nodes.

In our method, we employed hold-out validation to evaluate the performance of the model predictions. 80% of our training set is fed to the model, and the rest is used as a validation set. In training, the loss function is calculated by using multi-output binary cross entropy. Then, we employ the Rmsprop optimizer with the learning rate of 0.01 as well as the mini batch size of 64 to minimize the loss [71]. Initially, we initialize the weights of our deep neural model according to a uniform distribution [72]. To pick the model with best performance, we monitor the convergence of the loss function on the validation set and update the weights of the model after each training epoch. Finally, we employ dropout layers as regularizers to prevent our model from over-fitting. The source code and data of DeepMiR2GO are freely available at https://github.com/JChander/DeepMiR2GO.

#### 4.7.4. Evaluation Measures

Since the output of the hierarchical multi-classification model for each class is a prediction score between 0 and 1, we use a threshold value, denoted by *t*, to conclude the prediction results. For each threshold *t* ranging from 0 to 1, all GO terms with predicted scores greater than or equal to *t* are selected to make up the predicted set, which is indicated as P(t). In addition, we employ *R* to represent the set of GO terms determined by experimental validation. Three widely used statistical measurements are employed to assess the predictive performance of our model: precision, recall, and maximum F-measure [73,74]. For a given threshold *t* and miRNA *i*, the mathematical definitions of precision and recall are given as follows:

(4) $$\begin{array}{c} Pre_{i}\left(t\right)=\frac{\sum_{g}I\left(g\in P_{i}\left(t\right)⋀g\in R_{i}\right)}{\sum_{g}I\left(g\in P_{i}\left(t\right)\right)} \end{array}$$

(5) $$\begin{array}{c} Rec_{i}\left(t\right)=\frac{\sum_{g}I\left(g\in P_{i}\left(t\right)⋀g\in R_{i}\right)}{\sum_{g}I\left(g\in R_{i}\right)} \end{array}$$

Here, *g* denotes a specific GO class among the hierarchy, and I(x) denotes an indicator function stated as follows:

(6) $$I\left(x\right)=\left\{\begin{array}{cc} 1, & x=true\\ 0, & x=false \end{array}\right.$$

For each threshold *t*, the average precision is computed for the miRNAs which have at least one predicted term with a score higher than or equal to the threshold *t*, and m(t) represents the amount of these miRNAs. Similarly, we calculate the average recall and use *N* to denote the total number of all miRNAs in the benchmark. The definitions of the average precision and recall are as follows:

(7) $$\begin{array}{c} Pre\left(t\right)=\frac{1}{m(t)}\times \sum_{i=1}^{m(t)}Pre_{i}\left(t\right) \end{array}$$

(8) $$\begin{array}{c} Rec\left(t\right)=\frac{1}{N}\times \sum_{i=1}^{N}Rec_{i}\left(t\right) \end{array}$$

However, precision and recall are usually a pair of inversely related metrics. To handle the problem and utilize a single score to evaluate the performance, we calculate F-measure values for the threshold *t* ranging from 0 to 1 by combining average precision and average recall. Then, the maximum F-measure among all thresholds is selected.

(9) $$\begin{array}{c} F_{max}=max\left\{\frac{2\times Pre(t)\times Rec(t)}{Pre(t)+Rec(t)}\right\} \end{array}$$

## 5. Conclusions

In the past dozen years, large quantities of miRNAs have been identified. Accumulating evidence and studies have proven that miRNAs are involved in various essential biological processes and are associated with various diseases. However, the physiological functions of most miRNAs remain unknown. In this paper, we designed a novel method, DeepMiR2GO, based on multi-network convergence to annotate the ontological terms of miRNA by using a deep hierarchical classification model. Firstly, a global heterogeneous network is built by integrating the miRNA co-expression network, PPI network, disease similarity network, and network of associations among the three biological entities. Secondly, a network embedding approach, LINE, is applied for learning the low-dimensional topological features vector for each node of the global heterogeneous network. Then, a deep hierarchical multi-label classification model is constructed and trained with the low-dimensional network features. Finally, the miRNA2GO-337 dataset is used as an independent test set to assess the predictive performance. As a result, our DeepMiR2GO significantly outperforms two state-of-the-art approaches, miEAA and PmiRGO, in terms of three classic metrics: precision, recall, and $F_{max}$.

However, there is still room for improvement in the future. First, the miRNA co-expression network contains only a fraction of human miRNAs. More expression profiles of miRNA are expected to improve the performance of DeepMiR2GO. Next, the classification model of our work requires quite numerous training samples for each GO class, which are not available for prediction in other areas of application. Furthermore, we only focus on predicting functions of human miRNA: more species will be explored and applied to our model.

## Figures and Tables

**Table 1:** Predictive performance among different dimensions of network features for the miRNA2GO-337 dataset.

**Biological Processes**			
**Dimensionality**	$F_{\max}$	***AvePre***	***AveRec***
64	**0.399**	**0.404**	0.394
128	0.389	0.403	0.376
256	0.393	0.383	0.403
512	0.374	0.345	0.409
**Molecular Functions**			
**Dimensionality**	$F_{\max}$	***AvePre***	***AveRec***
64	**0.510**	0.500	**0.520**
128	0.399	0.376	0.424
256	0.504	0.510	0.498
512	0.411	0.470	0.364

AvePre denotes average precision, AveRec denotes average recall.

**Table 2:** Performance evaluation of the global network with and without the disease similarity network. The predictive performances were evaluated on the miRNA2GO-337 dataset.

**Biological Processes**			
**Dimensionality**	$F_{\max}$	***AvePre***	***AveRec***
With	**0.399**	**0.404**	**0.394**
Without	0.360	0.372	0.349
**Molecular Functions**			
**Dimensionality**	$F_{\max}$	***AvePre***	***AveRec***
With	**0.510**	**0.500**	**0.520**
Without	0.279	0.238	0.339

AvePre denotes average precision, AveRec denotes average recall.

**Table 3:** The top 17 GO terms of miRNA hsa-miR-92a-3p predicted by DeepMiR2GO.

ID	GO Terms	GO Names
1	GO:0044260	cellular macromolecule metabolic process
2	GO:0060255	regulation of macromolecule metabolic process
3	GO:0031323	regulation of cellular metabolic process
4	GO:0080090	regulation of primary metabolic process
5	GO:0043170	macromolecule metabolic process
6	GO:0050794	regulation of cellular process
7	GO:0050789	regulation of biological process
8	GO:0065007	biological regulation
9	GO:0044763	cellular process
10	GO:0071704	organic substance metabolic process
11	GO:0010468	regulation of gene expression
12	GO:0044237	cellular metabolic process
13	GO:0009987	cellular process
14	GO:0044238	primary metabolic process
15	GO:0019222	regulation of metabolic process
16	GO:0044699	biological process
17	GO:0008152	metabolic process

**Table 4:** The top 10 GO terms of miRNA hsa-miR-138-5p predicted by DeepMiR2GO. Depth denotes the number of layers of the miRNA in the DAG of GO.

ID	GO Terms	Depth	GO Names
1	GO:0045892	12	negative regulation of transcription
2	GO:0010629	7	negative regulation of gene expression
3	GO:0045944	13	positive regulation of transcription by RNA polymerase II
4	GO:1903507	11	negative regulation of nucleic acid-templated transcription
5	GO:1902679	10	negative regulation of RNA biosynthetic process
6	GO:0008285	6	negative regulation of cell population proliferation
7	GO:1903508	11	positive regulation of nucleic acid-templated transcription
8	GO:0000122	13	negative regulation of transcription by RNA polymerase II
9	GO:2000113	8	negative regulation of cellular
10	GO:0051253	9	negative regulation of RNA metabolic process

## Supplementary Materials

Supplementary Materials can be found at https://www.mdpi.com/1422-0067/20/23/6046/s1, Table S1: The miRNA2GO-337 dataset.

## Abbreviations

The following abbreviations are used in this manuscript:

PPI	Protein Protein Interaction
aa	Amino Acids
IEA	Inferred from Electronic Annotation
GO	Gene Ontology
BP	Biological Process
MF	Molecular Function
CC	Cellular Component
