# Supplementary material for: DeepMiR2GO: Inferring Functions of Human MicroRNAs Using a Deep Multi-Label Classification Model
Source: Int J Mol Sci. 2019 Nov 30;20(23):6046. doi: 10.3390/ijms20236046 (PMC6928926; doi:10.3390/ijms20236046)
Supplement: Supplementary file 1 [file ijms-20-06046-s001.pdf]

	gos	proteins
0	['GO:0005	hsa-miR-370-3p
1	['GO:0005	hsa-miR-941
2	['GO:0005	hsa-miR-19b-3p
3	['GO:0000	hsa-miR-33a-3p
4	['GO:0010	hsa-miR-29c-5p
5	['GO:0005	hsa-miR-421
6	['GO:0005	hsa-miR-532-3p
7	['GO:0035	hsa-miR-519b-3p
8	['GO:0005	hsa-miR-486-3p
9	['GO:0005	hsa-miR-424-3p
10	['GO:0005	hsa-miR-21-3p
11	['GO:0005	hsa-miR-4446-3p
12	['GO:0001	hsa-miR-1-3p
13	['GO:0010	hsa-miR-296-5p
14	['GO:0005	hsa-miR-7706
15	['GO:0005	hsa-miR-381-3p
16	['GO:0010	hsa-miR-494-3p
17	['GO:0001	hsa-miR-939-5p
18	['GO:0044	hsa-miR-339-5p
19	['GO:0005	hsa-miR-1307-5p
20	['GO:0000	hsa-miR-199a-3p
21	['GO:0005	hsa-miR-194-5p
22	['GO:0005	hsa-miR-340-5p
23	['GO:0005	hsa-miR-625-3p
24	['GO:0005	hsa-miR-185-5p
25	['GO:0035	hsa-miR-514a-3p
26	['GO:0001	hsa-miR-128-3p
27	['GO:0000	hsa-miR-145-5p
28	['GO:0035	hsa-miR-219a-5p
29	['GO:0005	hsa-miR-18a-5p
30	['GO:0035	hsa-miR-153-3p
31	['GO:0010	hsa-miR-206
32	['GO:0005	hsa-miR-132-3p
33	['GO:0005	hsa-miR-320b
34	['GO:0005	hsa-miR-431-5p
35	['GO:0035	hsa-miR-135b-5p
36	['GO:0008	hsa-miR-520a-3p
37	['GO:0005	hsa-miR-3173-5p
38	['GO:0005	hsa-miR-335-3p
39	['GO:0008	hsa-miR-515-3p
40	['GO:0035	hsa-miR-892b
41	['GO:0005	hsa-miR-99b-5p
42	['GO:0001	hsa-miR-29c-3p
43	['GO:0005	hsa-miR-574-3p
44	['GO:0005	hsa-miR-23b-3p
45	['GO:0001	hsa-miR-92a-3p
46	['GO:0005	hsa-miR-122-5p
47	['GO:0008	hsa-miR-711
48	['GO:0005	hsa-miR-204-5p
49	['GO:0005	hsa-miR-141-3p

50	['GO:0005(	hsa-miR-29b-3p
51	['GO:0003:	hsa-miR-195-5p
52	['GO:0005!	hsa-miR-518a-5p
53	['GO:0005(	hsa-miR-4286
54	['GO:0005(	hsa-miR-10a-5p
55	['GO:0005(	hsa-miR-423-3p
56	['GO:0005(	hsa-miR-106a-5p
57	['GO:0005(	hsa-miR-548o-3p
58	['GO:0005(	hsa-miR-192-5p
59	['GO:0005(	hsa-miR-215-5p
60	['GO:0035:	hsa-miR-873-5p
61	['GO:0005(	hsa-miR-199b-5p
62	['GO:0005(	hsa-miR-15b-3p
63	['GO:0005(	hsa-miR-24-3p
64	['GO:0030!	hsa-miR-1224-5p
65	['GO:0035:	hsa-miR-96-5p
66	['GO:0003(	hsa-miR-522-5p
67	['GO:0005(	hsa-miR-199a-5p
68	['GO:0005(	hsa-miR-664a-3p
69	['GO:0005(	hsa-miR-889-3p
70	['GO:0005(	hsa-miR-342-5p
71	['GO:0035:	hsa-miR-520b
72	['GO:0005(	hsa-miR-222-3p
73	['GO:0035:	hsa-miR-499a-3p
74	['GO:0010(	hsa-miR-132-5p
75	['GO:0010!	hsa-miR-196a-5p
76	['GO:0000!	hsa-miR-584-5p
77	['GO:0005(	hsa-miR-148b-3p
78	['GO:0005!	hsa-miR-518d-5p
79	['GO:0005(	hsa-miR-181a-3p
80	['GO:0005:	hsa-miR-92a-1-5p
81	['GO:0005(	hsa-miR-339-3p
82	['GO:0005(	hsa-miR-26b-5p
83	['GO:0005(	hsa-miR-27a-3p
84	['GO:0005(	hsa-miR-937-3p
85	['GO:0005(	hsa-miR-487b-3p
86	['GO:0005(	hsa-miR-345-5p
87	['GO:0010(	hsa-miR-548c-3p
88	['GO:0001!	hsa-miR-21-5p
89	['GO:0000!	hsa-miR-1-5p
90	['GO:0030{	hsa-miR-18b-5p
91	['GO:0005(	hsa-miR-99a-5p
92	['GO:0005(	hsa-miR-100-5p
93	['GO:0005(	hsa-miR-363-3p
94	['GO:1903(	hsa-miR-377-3p
95	['GO:0005(	hsa-miR-30c-5p
96	['GO:0005(	hsa-miR-425-5p
97	['GO:0005(	hsa-miR-590-5p
98	['GO:0010(	hsa-miR-34a-5p
99	['GO:0005(	hsa-miR-205-5p
100	['GO:0005(	hsa-miR-130b-3p

101	['GO:0005	hsa-miR-126-3p
102	['GO:0005	hsa-miR-151a-5p
103	['GO:0035	hsa-miR-200a-3p
104	['GO:0002	hsa-miR-155-5p
105	['GO:0005	hsa-miR-221-3p
106	['GO:0005	hsa-miR-1296-5p
107	['GO:0005	hsa-miR-23a-3p
108	['GO:0005	hsa-miR-144-5p
109	['GO:0005	hsa-miR-15a-5p
110	['GO:0005	hsa-miR-24-2-5p
111	['GO:0005	hsa-miR-28-3p
112	['GO:0005	hsa-miR-181a-5p
113	['GO:0005	hsa-miR-151b
114	['GO:0002	hsa-miR-208a-3p
115	['GO:0035	hsa-miR-663a
116	['GO:0005	hsa-miR-501-3p
117	['GO:0005	hsa-miR-214-3p
118	['GO:0035	hsa-miR-193a-3p
119	['GO:0005	hsa-miR-451a
120	['GO:0005	hsa-miR-221-5p
121	['GO:0005	hsa-miR-378a-3p
122	['GO:0005	hsa-miR-409-3p
123	['GO:0005	hsa-miR-18a-3p
124	['GO:0005	hsa-miR-1468-5p
125	['GO:0005	hsa-miR-3615
126	['GO:0005	hsa-miR-130a-3p
127	['GO:0005	hsa-miR-4732-3p
128	['GO:0005	hsa-miR-411-5p
129	['GO:0000	hsa-miR-139-5p
130	['GO:0005	hsa-miR-16-2-3p
131	['GO:0048	hsa-miR-17-3p
132	['GO:0008	hsa-miR-1290
133	['GO:0005	hsa-miR-1249-3p
134	['GO:0000	hsa-miR-375
135	['GO:0005	hsa-miR-323b-3p
136	['GO:0005	hsa-miR-4732-5p
137	['GO:0005	hsa-miR-1273h-3p
138	['GO:0005	hsa-miR-323a-3p
139	['GO:0010	hsa-miR-548p
140	['GO:0005	hsa-miR-25-5p
141	['GO:0005	hsa-miR-31-5p
142	['GO:0010	hsa-miR-509-3p
143	['GO:0016	hsa-miR-193a-5p
144	['GO:0005	hsa-miR-223-5p
145	['GO:0005	hsa-miR-191-5p
146	['GO:0005	hsa-miR-6087
147	['GO:0014	hsa-miR-499a-5p
148	['GO:1905	hsa-miR-34a-3p
149	['GO:0035	hsa-miR-639
150	['GO:0005	hsa-miR-26b-3p
151	['GO:0005	hsa-miR-1307-3p

152	['GO:0008	hsa-miR-372-3p
153	['GO:0035	hsa-miR-200c-3p
154	['GO:0005	hsa-miR-224-5p
155	['GO:0000	hsa-miR-20a-5p
156	['GO:0035	hsa-miR-34c-5p
157	['GO:0001	hsa-miR-30b-5p
158	['GO:0005	hsa-miR-769-5p
159	['GO:0005	hsa-miR-148a-3p
160	['GO:0005	hsa-miR-181d-5p
161	['GO:0035	hsa-miR-495-3p
162	['GO:0005	hsa-miR-140-5p
163	['GO:0035	hsa-miR-181b-3p
164	['GO:0005	hsa-miR-93-5p
165	['GO:0035	hsa-miR-362-3p
166	['GO:0005	hsa-miR-3158-3p
167	['GO:0005	hsa-miR-142-5p
168	['GO:0005	hsa-miR-671-3p
169	['GO:0005	hsa-miR-374b-5p
170	['GO:0035	hsa-miR-483-5p
171	['GO:0005	hsa-miR-5189-5p
172	['GO:0005	hsa-miR-485-5p
173	['GO:0003	hsa-miR-518c-5p
174	['GO:0005	hsa-miR-208b-3p
175	['GO:0005	hsa-miR-7-5p
176	['GO:0005	hsa-miR-4433b-5p
177	['GO:0005	hsa-miR-410-3p
178	['GO:0005	hsa-miR-486-5p
179	['GO:0005	hsa-miR-182-5p
180	['GO:0005	hsa-miR-148b-5p
181	['GO:0005	hsa-miR-98-5p
182	['GO:0005	hsa-miR-4508
183	['GO:0005	hsa-miR-652-3p
184	['GO:0000	hsa-miR-125a-5p
185	['GO:0035	hsa-miR-32-3p
186	['GO:0005	hsa-miR-518b
187	['GO:0005	hsa-miR-638
188	['GO:0005	hsa-miR-589-5p
189	['GO:0005	hsa-miR-183-5p
190	['GO:0003	hsa-miR-1825
191	['GO:0000	hsa-miR-16-5p
192	['GO:0005	hsa-miR-766-3p
193	['GO:0005	hsa-miR-331-3p
194	['GO:0005	hsa-miR-28-5p
195	['GO:0005	hsa-miR-101-3p
196	['GO:0005	hsa-miR-181c-3p
197	['GO:0003	hsa-miR-15b-5p
198	['GO:0003	hsa-miR-17-5p
199	['GO:0035	hsa-miR-519e-3p
200	['GO:0005	hsa-miR-29a-3p
201	['GO:0005	hsa-miR-361-5p
202	['GO:0000	hsa-miR-297

203	['GO:0005	hsa-miR-106b-5p
204	['GO:0035	hsa-miR-299-5p
205	['GO:0005	hsa-miR-151a-3p
206	['GO:0005	hsa-miR-210-3p
207	['GO:0001	hsa-miR-146a-5p
208	['GO:0035	hsa-miR-572
209	['GO:0010	hsa-miR-492
210	['GO:1904	hsa-miR-214-5p
211	['GO:0005	hsa-miR-636
212	['GO:0005	hsa-miR-186-5p
213	['GO:0005	hsa-miR-532-5p
214	['GO:0035	hsa-miR-543
215	['GO:0051	hsa-miR-34b-5p
216	['GO:0005	hsa-miR-518c-3p
217	['GO:0005	hsa-miR-19a-3p
218	['GO:0035	hsa-miR-590-3p
219	['GO:0005	hsa-miR-30a-3p
220	['GO:0035	hsa-miR-329-3p
221	['GO:0005	hsa-miR-25-3p
222	['GO:0035	hsa-miR-876-3p
223	['GO:0005	hsa-miR-197-3p
224	['GO:0005	hsa-miR-1306-5p
225	['GO:0005	hsa-miR-320a
226	['GO:0002	hsa-miR-328-3p
227	['GO:0003	hsa-miR-518d-3p
228	['GO:0005	hsa-miR-432-5p
229	['GO:0003	hsa-miR-518e-3p
230	['GO:0005	hsa-miR-500a-3p
231	['GO:0005	hsa-miR-874-3p
232	['GO:0000	hsa-miR-378a-5p
233	['GO:0005	hsa-miR-134-5p
234	['GO:0010	hsa-miR-124-3p
235	['GO:0005	hsa-miR-92b-3p
236	['GO:0005	hsa-miR-125b-2-3p
237	['GO:0035	hsa-miR-661
238	['GO:0005	hsa-miR-505-3p
239	['GO:0035	hsa-miR-448
240	['GO:0005	hsa-miR-185-3p
241	['GO:0005	hsa-miR-5010-5p
242	['GO:0001	hsa-miR-424-5p
243	['GO:0005	hsa-miR-146b-5p
244	['GO:0005	hsa-miR-340-3p
245	['GO:0035	hsa-miR-301b-3p
246	['GO:0035	hsa-miR-2355-5p
247	['GO:0005	hsa-miR-146b-3p
248	['GO:0008	hsa-miR-519d-3p
249	['GO:0005	hsa-miR-107
250	['GO:0005	hsa-miR-181a-2-3p
251	['GO:0005	hsa-miR-93-3p
252	['GO:0035	hsa-miR-665
253	['GO:0005	hsa-miR-150-3p

254	['GO:0005	hsa-miR-550a-3p
255	['GO:0005	hsa-miR-1301-3p
256	['GO:0005	hsa-miR-628-3p
257	['GO:0006	hsa-miR-15a-3p
258	['GO:0005	hsa-miR-374a-5p
259	['GO:0005	hsa-miR-130b-5p
260	['GO:0000	hsa-miR-140-3p
261	['GO:0005	hsa-miR-493-5p
262	['GO:0035	hsa-miR-129-5p
263	['GO:0001	hsa-miR-103a-3p
264	['GO:0005	hsa-miR-142-3p
265	['GO:0010	hsa-miR-133b
266	['GO:0005	hsa-miR-27b-3p
267	['GO:0005	hsa-miR-143-3p
268	['GO:0007	hsa-miR-9-5p
269	['GO:0007	hsa-miR-675-5p
270	['GO:0005	hsa-miR-877-5p
271	['GO:0035	hsa-miR-582-5p
272	['GO:0001	hsa-miR-22-3p
273	['GO:0016	hsa-miR-217
274	['GO:0035	hsa-miR-188-5p
275	['GO:0035	hsa-miR-145-3p
276	['GO:0005	hsa-miR-576-5p
277	['GO:0035	hsa-miR-200c-5p
278	['GO:0005	hsa-miR-7849-3p
279	['GO:0005	hsa-miR-3605-3p
280	['GO:0003	hsa-miR-518a-3p
281	['GO:0005	hsa-miR-6511a-3p
282	['GO:0005	hsa-miR-30e-5p
283	['GO:0005	hsa-miR-423-5p
284	['GO:0035	hsa-miR-767-5p
285	['GO:0005	hsa-miR-6511b-3p
286	['GO:0005	hsa-miR-10b-5p
287	['GO:0005	hsa-miR-127-3p
288	['GO:0005	hsa-miR-191-3p
289	['GO:0005	hsa-miR-320c
290	['GO:0005	hsa-miR-374a-3p
291	['GO:0035	hsa-miR-657
292	['GO:0005	hsa-miR-744-5p
293	['GO:0005	hsa-miR-26a-5p
294	['GO:0005	hsa-miR-342-3p
295	['GO:0005	hsa-miR-150-5p
296	['GO:0005	hsa-miR-505-5p
297	['GO:0005	hsa-miR-181c-5p
298	['GO:0005	hsa-miR-660-5p
299	['GO:0005	hsa-miR-181b-5p
300	['GO:0005	hsa-miR-338-3p
301	['GO:0007	hsa-miR-137
302	['GO:0035	hsa-miR-497-5p
303	['GO:0005	hsa-miR-484
304	['GO:0005	hsa-miR-4632-3p

305	['GO:0016	hsa-miR-10a-3p
306	['GO:0010	hsa-miR-640
307	['GO:0005	hsa-miR-30a-5p
308	['GO:0005	hsa-miR-144-3p
309	['GO:0005	hsa-miR-125b-5p
310	['GO:0005	hsa-miR-20b-5p
311	['GO:0005	hsa-miR-376c-3p
312	['GO:0005	hsa-miR-301a-3p
313	['GO:0005	hsa-miR-133a-3p
314	['GO:0005	hsa-miR-454-3p
315	['GO:0005	hsa-miR-30e-3p
316	['GO:0008	hsa-miR-218-5p
317	['GO:0008	hsa-miR-520h
318	['GO:0005	hsa-miR-335-5p
319	['GO:0005	hsa-miR-361-3p
320	['GO:0035	hsa-miR-298
321	['GO:0000	hsa-miR-126-5p
322	['GO:0005	hsa-miR-138-5p
323	['GO:0005	hsa-miR-502-3p
324	['GO:0003	hsa-miR-518f-3p
325	['GO:0005	hsa-miR-152-3p
326	['GO:0003	hsa-miR-518f-5p
327	['GO:0001	hsa-miR-503-5p
328	['GO:0008	hsa-miR-200b-3p
329	['GO:0005	hsa-miR-654-3p
330	['GO:0035	hsa-miR-483-3p
331	['GO:0005	hsa-miR-106b-3p
332	['GO:0005	hsa-miR-223-3p
333	['GO:0005	hsa-miR-136-3p
334	['GO:0005	hsa-miR-212-3p
335	['GO:0005	hsa-miR-30d-5p
336	['GO:0035	hsa-miR-105-5p
